# Electrocardiographic changes in therapeutic hypothermia

**DOI:** 10.1186/cc11369

**Published:** 2012-06-06

**Authors:** Corina L Rolfast, Erik J Lust, Carel C de Cock

**Affiliations:** 1 Department of Cardiology and Institute for Cardiovascular Research VU, VU University Medical Center, De Boelelaan 1117, 1081 HV Amsterdam, The Netherlands

## Abstract

**Introduction:**

During therapeutic hypothermia (TH), electrocardiographic (ECG) abnormalities such as Osborn waves and/or ST-segment elevation have been described. However, the incidence and prognostic value of these ECG changes are uncertain given the small-scale studies that have been carried out to date. The aim of this study is to further evaluate the electrocardiographic changes during TH.

**Methods:**

During a period of 3 years, 81 patients (age 63 ± 14 years) were included retrospectively. All patients underwent TH after being resuscitated. ECG registrations before, during and after TH were collected and analyzed. Patients were divided into two groups based on the presence or absence of transmural ischemia ST elevation on the first representative ECG upon arrival at the hospital (ST-segment elevation myocardial infarction (STEMI) and non-STEMI).

**Results:**

A total of 243 ECGs were analyzed. During TH 24 patients (30%) had Osborn waves, which disappeared in 22 patients (92%) after regaining normal body temperature. The presence of Osborn waves was not associated with age, gender, average pH, electrolytes, or lactate levels and was not associated with excess in-hospital mortality. In 10 patients (12%, six non-STEMI patients) new STEMI was observed during TH, which disappeared after TH discontinuation. The STEMI group (44 patients) had significantly more Osborn waves during TH than the non-STEMI group (38.6% vs. 15.2%, odds ratio = 3.508; 95% confidence interval = 1.281 to 9.610).

**Conclusions:**

Hypothermia-induced Osborn waves are relatively common and are not associated with an unfavorable short-term outcome. TH is associated with ECG changes that may mimic STEMI.

## Introduction

Post-anoxic encephalopathy is a common complication after an out-of-hospital cardiac arrest (OHCA). The risk of neurological damage remains high even after being successfully resuscitated [1-5]. Hours and days after successful resuscitation, further damage can be caused by multiple deployed cascades - such as excitotoxicity by the release of certain amino acids and free oxygen radicals that trigger a chemical reaction leading to further DNA damage and apoptosis of brain cells [6,7].

The current guidelines recommend mild therapeutic hypothermia (TH) to prevent neurological damage after a cardiac arrest. Hypothermia is defined as a body temperature <35°C [8]. Using mild temperatures of 32 to 33°C, relatively few side effects have been reported. A number of studies have conclusively shown that mild hypothermia significantly reduced the risk of hypoxic brain damage and has a beneficial effect on short-term survival [9-11]. Although the pathophysiology underlying these results has insufficiently been clarified, applying mild hypothermia after resuscitation has become a standard of care worldwide.

Hypothermia also has an influence on the conduction of the heart. Some small studies have shown that hypothermia leads to electrocardiographic (ECG) changes including Osborn waves [12,13]. Several case reports describe abnormalities during TH that may be indicative of cardiac ischemia, particularly ST-segment elevation myocardial infarction (STEMI) [14-17]. However, these studies were too small to make clear conclusions on the incidence and prognostic value of these changes. The aim of the present study is to further evaluate the electrocardiographic changes during TH after OHCA in a larger cohort and to study whether these changes were associated with an adverse outcome.

## Materials and methods

The study was approved by the VU University Medical Center institutional review board (number R 2011/161, IRB 00002991. In the period of January 2007 to October 2009 TH was applied to 109 consecutive patients. By protocol, midazolam and propovol were used for sedation. A 12-lead ECG was collected from all patients in addition to clinical and laboratory parameters.

Patients were included if one or more ECGs with the patient's corresponding body temperature before, during and after TH were present. Patients with a complete left bundle branch block (11 patients) and patients with a pacemaker rhythm (2 patients) were excluded. ECG registration was incomplete in 15 patients and these patients were also excluded. Eighty-one patients were thus included and 243 ECGs were analyzed.

All ECGs were assessed by two experienced cardiologists (CDC, EJL). During TH a temperature averaging 32°C (minimum 29°C, maximum 34°C) was maintained for 12 to 24 hours. The presenting arrhythmia was defined as that initially recorded by the emergency medical services, and this was judged by two cardiologists (CCdC, EJL). When the initial strip was not available, the rhythm was identified from the emergency medical services report. Ventricular fibrillation (VF) was defined as a pulseless condition with specific features on the cardiac recording, while pulseless electrical activity was defined as the absence of a pulse with the appearance of an organized electrical rhythm [18].

Patients were divided into two groups, based on the presence or absence of signs of acute transmural ischemia on the 12-lead ECG on admission: STEMI group and non-STEMI group. Patients with ST segment elevation ≥1 mm in ≥2 contiguous leads were considered to have STEMI, and these patients underwent emergency coronary angiography and primary percutaneous coronary intervention [19]. In the non-STEMI patient group, a different cause than coronary artery disease was assumed based on the clinical and electrocardiographic data upon arrival at the hospital. In these patients, no emergency coronary angiography was performed. We did not use the cardiac enzymes as markers for ischemia, considering there are conflicting data on the sensitivity and specificity of these markers in these patients to assess myocardial infarction [20].

The following parameters were analyzed before, during and after TH: heart rate, basic heart rhythm, PQ conduction time, QRS conduction time, QTc time, left anterior fascicular block, Osborn waves, pathological Q waves and ST-T segment abnormalities. Q and QS patterns were scored according to the Minnesota Code Classification System [20]. QTc intervals were calculated as described by Bossaert and colleagues [21]. The following arrhythmias were scored; atrial fibrillation, atrioventricular nodal rhythm, atrioventricular block, ventricular tachycardia, ventricular.

### Statistical analysis

All statistical analyses were carried out using SPSS (SPSS Inc, Chicago, Illinois, USA). Results with *P *<0.05 were regarded as statistically significant. When comparing two discrete variables, the chi-square test or the odds ratio (OR) was used. We used a paired *t *test when comparing two groups of patients. For assessment of a linear relationship between a continuous variable and a dichotomous variable, the chi-square test for trend was used.

## Results

Table 1 presents the demographics of the 81 patients included. The mean age of the patients was 63 ± 11 years and 74% were male. In all patients the onset of the cardiac arrest was outside the hospital. In 56 patients (69%) the initial rhythm was VF. In the remaining group, 19 patients (24%) presented with asystole and five patients (6%) had pulseless electrical activity. VF was more commonly present in the STEMI group (OR = 6.333; 95% confidence interval (CI) = 2.058 to 19.487). Acute STEMI was considered the cause of OHCA in 54 patients (56%). The in-hospital mortality in the total group was 50%. From the group of patients that survived, 38 patients (47%) were diagnosed to have moderate to severe post-anoxic encephalopathy. Survival to discharge was substantially more likely after documented VF than after documented non-VF (68% vs. 12%, OR = 15; 95% CI = 4.093 to 58.552).

**Table 1:** Demographic factors in the total study cohort

Age (years)	63 ± 14
Male/female	60 (74%)/21 (26%)
Index arrhythmia	
Ventricular fibrillation	56 (69%)
Nonventricular fibrillation (pulseless electrical activity/asystole)	25 (31%)
Heart rhythm at hospital arrival	
Sinus rhythm	56 (69%)
Atrial fibrillation	18 (22%)
Atrial flutter	1 (1%)
Atrioventricular nodal rhythm	6 (8%)
Underlying condition	
Myocardial infarction	45 (56%)
Asphyxia/aspiration	5 (6%)
Pulmonary embolism	5 (6%)
Chronic obstructive pulmonary disease	2 (3%)
Brugada syndrome	1 (1%)
Myocarditis	1 (1%)
Severe aortic valve stenosis	3 (4%)
Advanced heart failure	2 (3%)
Unknown	17 (21%)

Data presented as mean ± standard deviation or *n *(%).

All 243 ECG tracings were assessed for the presence of TH-induced electrocardiographic changes (Table 2). The heart rate showed a consistent reduction from 95 ± 15 to 58 ± 13 beats/minute (39% reduction, *P *<0.0001), and QTc showed a significant 7% increase (466 ± 54 milliseconds before TH to 498 ± 65 milliseconds during TH, *P *<0.0001). The ECG before initiating TH showed no Osborn waves in any patient. Osborn waves occurred in 24 patients (30%) during TH. In 22 of these patients, the Osborn waves disappeared after regaining normal body temperature. A direct correlation between the temperature during TH and the emergence of Osborn waves was not observed (*P *= 0.382). Osborn waves were significantly more prevalent during cooling in the STEMI group than in the non-STEMI group (38.6% vs. 15.2%, OR = 3.508, 95% CI = 1.281 to 9.610). The presence of Osborn waves was not associated with an increase in in-hospital mortality (50.9% vs. 45.8%, OR = 0.817, 95% CI = 0.314 to 2.12). TH-induced Osborn waves also showed no relation to age or gender, and potassium and calcium levels did not correlate with any ECG changes. The two patients with persistent Osborn waves both survived until hospital discharge.

**Table 2:** Electrocardiographic changes and arrhythmia before, during and after therapeutic hypothermia

	Before TH	During TH	After TH
Heart rate (beats/minute)	95 ± 26	58 ± 15	87 ± 17
PQ duration (milliseconds)	172 ± 45	175 ± 44	155 ± 34
QRS duration (milliseconds)	103 ± 28	99 ± 21	89 ± 18
QTc (milliseconds)	466 ± 54	498 ± 65	460 ± 55
LAFB	14	7	6
Osborn waves	0	24	2
ST elevation	38	22	12
ST depression	79	74	68
Right bundle branch block	7	9	8
Left bundle branch block	0	1	0
Q wave	15	23	21
Atrial fibrillation	16	4	4
Atrioventricular nodal rhythm	6	3	3
Ventricular tachycardia/ventricular fibrillation	6	1	0

Data presented as mean ± standard deviation or *n *(%). TH, therapeutic hypothermia.

**Figure 1:**
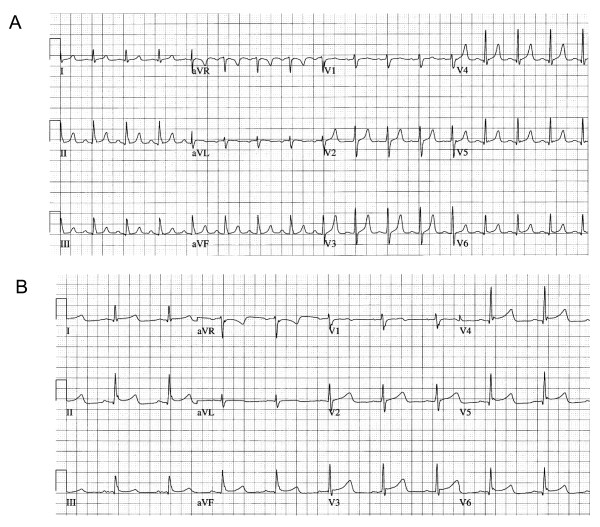
**Electrocardiogram changes before and during therapeutic hypothermia**. Example of electrocardiogram changes **(A) **before and**(B) **during therapeutic hypothermia in an 18-year old out-of-hospital cardiac arrest patient.

The average pH level at admission was 7.09 ± 0.18, ranging from 6.67 ± 0.12 to 7.39 ± 0.20. The average lactate concentration at admission was 6.93 ± 2.1 mmol/l, ranging from 0.50 ± 0.12 to 51.4 ± 16.0 mmol/l. A correlation between pH and lactate levels at admission and Osborn waves during cooling was not observed (*P *= 0.651 and *P *= 0.187, respectively). Of the 37 patients in the non-STEMI group, six patients developed new ST elevation during TH (Table 2). Two of the six patients were younger than 35 years old without a cardiac history (see Figure 1). As a result of the ST-segment changes during TH, two patients underwent emergency coronary angiography showing normal epicardial coronary arteries. In five of the six patients, the ST segment elevation disappeared after TH.

In addition, four patients in the STEMI group developed new ST elevation during TH (Table 2). Two of the four patients had a proven acute anterior wall infarction and developed new ST-segment elevation in the inferior leads during TH. The remaining two patients had a proven inferior wall infarction and developed new ST-segment elevation in the anterior leads. Of interest, there was no reciprocal depression corresponding with the new ST elevations during TH. In two of the four patients, the ECG tracing after TH showed ST-segment elevation in the leads that represented the myocardial infarction. In the six patients classified as nonischemic, all had echocardiography to assess the need for coronary intervention. Global and/or regional wall motion abnormalities were observed in two patients, who subsequently underwent emergency coronary angiography showing no abnormalities. In none of the patients was a percutaneous coronary intervention performed. The new ST elevation that developed during TH disappeared in all patients after TH. Eight patients developed a pathological Q wave during cooling, while there were no pathological Q waves before TH. All eight of these patients, however, were classified as the STEMI group before TH initiation.

## Discussion

The Advanced Life Support Task Force of the International Liaison Committee on Resuscitation published guidelines on OHCA patients, recommending cooling to 32 to 34°C for 12 to 24 hours in 2003 [22]. TH has been rapidly implemented since then, with the number of ICUs using this therapy reported to be up to 95% [23]. Although ECG abnormalities associated with hypothermia were described in 1938, remarkably few studies including a limited number of patients have focused on the prevalence and possible predictive value of these abnormalities [12-17].

Rankin and Rae studied 22 patients with accidental hypothermia and observed Osborn waves in one-half of the study cohort [12]. The mean temperature was low (29.8 ± 3.3°C) as compared with TH, however, and in 50% of patients the temperature on admission was **<**30°C. Atrial fibrillation was seen in 27% and QTc prolongation in 90% of patients. Mortality was high (64%) and a relation with outcome of ECG abnormalities or arrhythmias was not reported [12].

Vassallo and colleagues studied 100 ECGs in 43 consecutive patients with accidental hypothermia [14]. Presenting temperatures were low, with 63% of patients ranging from 31.7 to 26.1°C. Osborn waves were present in all ECGs where the temperature was ≤30.5°C. Changes in QTc were not reported, and atrial fibrillation was seen in 19% of patients. Okada and colleagues studied 50 patients with accidental hypothermia and reported Osborn waves in 80% of patients [13]. The presence and size of the Osborn wave were inversely related to body temperature, and in three patients persistent Osborn waves were seen after prolonged hypothermia. The same group studied the incidence of arrhythmias during accidental hypothermia and observed atrial fibrillation in 23 patients (38%). A substantial number (70%) had a presenting body temperature **<**32º C. No significant difference in mortality was observed between patients with or without atrial fibrillation during hypothermia [13].

No studies on ECG abnormalities and arrhythmias during TH have been published. The main finding of the present study is that Osborn waves are a relatively common finding in OHCA patients receiving TH, being observed in 30% of all patients. The prevalence of TH-associated Osborn waves is substantially lower as compared with studies on patients with accidental hypothermia and is in concordance with earlier observations that these ECG abnormalities are inversely correlated with temperature [14]. In our study, the temperature maintained during TH was on average 32°C and 86.4% of the study cohort had a body temperature between 32 and 34°C during TH. The presence of Osborn waves was not associated with a higher in-hospital mortality. TH-induced Osborn waves were not correlated with age, gender, average pH, electrolytes or lactate levels on admission. Osborn waves were significantly more present in the ischemic group than in the nonischemic group (38.6% vs. 15.2%, OR = 3.508, 95% CI = 1.281 to 9.610). In 22 of the 24 patients, the Osborn waves disappeared spontaneously after TH. This suggests that Osborn waves are not an expression of cardiac structural changes occurring during hypothermia. Persistence of Osborn waves was also reported by Vassallo and coworkers and might reflect underlying structural heart disease [14].

A significant increase in QTc during TH was observed, which is in concordance with earlier studies. QRS duration was not influenced by TH. The most common arrhythmia during TH is atrial fibrillation, which has been reported to range from 19 to 38%. In our study, atrial fibrillation was seen in 18 patients (22%) and no association with the presence of atrial fibrillation and in-hospital mortality was found. ST-segment abnormalities are almost always present in up to 98% in our series. TH-associated ST-segment elevation has occasionally been reported in earlier studies and can mimic acute myocardial infarction [17]. Misdiagnosis in these cases can lead to inappropriate administration of thrombolytic therapy or unnecessary transport of these critically ill patients to a catheterization room. In addition, these patients can be subjected to nephrotoxic agents during coronary angiography.

Characteristics of the 10 patients in the present study who developed ST-segment elevation (12%) are shown in Table 3. Reciprocal ST-segment depression was not observed in any of these cases. Six out of the 10 patients with new ST elevation developing during TH belonged to the non-STEMI group. However, three of the six patients had a history of myocardial infarction. In the ischemic group, two patients with angiographically proven inferior infarction developed new ST-segment elevation during TH in the anterior leads, whereas two patients with an angiographically proven anterior STEMI had new ST elevations in the inferior leads. TH-induced ST-segment elevation disappeared in all patients after regaining normal body temperature. Reciprocal ST-segment depression in STEMI is often considered to be mirror-image changes or remote ischemia in a distant territory in patients with multivessel coronary artery disease [24,25]. The observed ST-segment elevation during TH probably reflects a different mechanism where heterogeneity in repolarization leads to a current flowing from the normal myocardium towards an area with the short action potential [26]. This mechanism may potentially lead to re-entrant tachycardia, but this was not observed in the present cohort.

**Table 3:** Characteristics of patients that developed ST-segment elevation during therapeutic hypothermia

	ST-segment elevation						
					
Patient	Before TH	New during TH	After TH	Diagnosis at admission	Cardiac history	Age (years)	Gender
Nonischemic							
1	0	AW = 4, IW = 2	0	Unknown	Hypertension	72	Female
2	0	AW = 4	0	Asphyxia	COPD	25	Male
3	0	AW = 2	0	Unknown	CABG	67	Male
4	0	AW = 2	0	Aspiration	-	34	Male
5	0	IW = 2	0	Unknown	Old MI	52	Male
6	0	AW = 2	AW = 2	Aspiration	CAD	59	Female
Ischemic							
1	IW = 0, AW = 4	IW = 3, AW = 3	IW = 0, AW = 3	Acute AW infarction	Hypertension	65	Female
2	IW = 0, AW = 5	IW = 3, AW = 5	0	Acute AW infarction	-	50	Female
3	IW = 3, AW = 0	IW = 2, AW = 4	0	Acute IW infarction	-	58	Male
4	IW = 3, AW = 0	IW = 3, AW = 2	IW = 2, AW = 0	Acute IW infarction	Old MI	47	Male

AW, anterior wall; CABG, coronary artery bypass graft; CAD, coronary artery disease; COPD, chronic obstructive pulmonary disease; IW, inferior wall; MI, myocardial infarction; TH, therapeutic hypothermia; VF, ventricular fibrillation.

### Limitations

Some important limitations have to be discussed. Although this is to date the largest study evaluating ECG findings in hypothermic patients, absolute numbers in this retrospective analysis are relatively small. In addition, comparison with studies on accidental hypothermia is ambiguous since body temperature in accidental hypothermia is substantially lower as compared with TH. Finally, patients were divided into STEMI and non-STEMI etiology of OHCA based on the first 12-lead ECG, which may not always reflect the true underlying pathophysiological substrate.

## Conclusion

TH is not uncommonly associated with abnormal ECG findings such as Osborn waves, prolongation of the QTc interval and atrial fibrillation, which have no impact on in-hospital outcome. TH-induced ST-segment elevation mimicking STEMI was seen in 12% of all patients. Absence of reciprocal ST-segment depression may be indicative of pseudo-infarction, which may prevent inappropriate or hazardous therapy.

## Key messages

• Osborne waves are the most common ECG changes during TH, observed in almost one-third of patients.

• The presence of Osborne waves is not associated with adverse in-hospital outcome.

• ST-segment elevation during hypothermia was seen in 12% of patients and may mimic acute myocardial infarction; however, unlike acute infarction, an absence of reciprocal ST depression was found in all patients.

## Abbreviations

CI: confidence interval; ECG: electrocardiogram; OHCA: out-of-hospital cardiac arrest; OR: odds ratio; STEMI: ST-segment elevation myocardial infarction; TH: therapeutic hypothermia; VF: ventricular fibrillation.

## Competing interests

The authors declare that they have no competing interests.

## Authors' contributions

CLR carried out the study, collected all data including ECG tracings and blood analysis, performed the statistical analysis and drafted the manuscript. EJL and CCdC supervised the conduct of the study and writing of the paper and performed ECG tracing analysis. All authors read and approved the final manuscript.

## References

[B1] Horsted TI Rasmussen LS Lippert FK Nielsen SL Outcome of out-of hospital cardiac arrest - why do physicians withhold resuscitation attempts? Resuscitation 2004 63 287 293 10.1016/j.resuscitation.2004.05.005 15582764

[B2] Rudner R Jalowiecki P Karpel E Dziurdzik P Alberski B Kawecki P Survival after cardiac arrest in Katowice (Poland): outcome report according to the 'Utstein style' Resucitation 2004 61 315 325 10.1016/j.resuscitation.2004.01.020 15172711

[B3] Fredriksson M Herlitz J Engdahl J Nineteen years experience of out-of-hospital cardiac arrest in Gothenburg - reported in Utstein style Resuscitation 2003 58 37 47 10.1016/S0300-9572(03)00115-1 12867308

[B4] Bunch TJ White RD Gersh BJ Meverden RA Hodge DO Ballman KV Hammill SC Shen WK Packer DL Long-term outcomes of out-of-hospital cardiac arrest after succesful early defibrillation N Engl J Med 2003 348 2626 2633 10.1056/NEJMoa023053 12826637

[B5] Herlitz J Bang A Gunnarsson J Engdahl J Karlson BW Lindqvist J L Waagstein Factors associated with survival to hospital discharge among patients hospitalized alive after out-of-hospital cardiac arrest Heart 2003 89 25 30 10.1136/heart.89.1.25 12482785 PMC1767484

[B6] Hampton T Researchers warm up to hypothermia after cardiac and brain trauma JAMA 2007 298 1994 1995 17986687 10.1001/jama.298.17.1994

[B7] Ridenour TR Warner DS Todd MM McAllister AC Mild hypothermia reduces infarct size from temporary but not permanent focal ischemia in rats Stroke 1992 23 733 738 10.1161/01.STR.23.5.733 1579972

[B8] Nolan JP Soar J Zideman DA Biarent D Bossaert LL Deakin C Koster RW Wyllie J Böttiger B ERC Guidelines Writing Group Council Guidelines for Resuscitation 2010. Section 1: executive summary Resuscitation 2010 81 1219 1276 10.1016/j.resuscitation.2010.08.021 20956052

[B9] Tianen M Poutiainen E Kovola T Takkunen O Häppölä O Roine RO Cognitive and neurophysiological outcome of cardiac arrest survivors treated with therapeutic hypothermia Stroke 2007 38 2303 2308 10.1161/STROKEAHA.107.483867 17585081

[B10] Kim F Olsufka M Longstreth WT Jr Maynard C Carlbom D Deem S Kudenchuk P Copass MK Cobb LA Pilot randomized clinical trial of prehospital induction of mild hypothermia in out-of-hospital cardiac arrest patients with a rapid infusion of 4°C normal saline Circulation 2007 115 3064 3070 10.1161/CIRCULATIONAHA.106.655480 17548731

[B11] Kämäräinen A Virkkunen I Tenhunen J YliHankala A Silvast P Prehospital induction of therapeutic hypothermia during CPR; a pilot study Resuscitation 2008 6 360 363 17936493 10.1016/j.resuscitation.2007.08.015

[B12] Rankin AC Rae A Cardiac arrhythmias during rewarming of patients with accidental hypothermia Br Med J 1984 289 874 877 10.1136/bmj.289.6449.874 6434119 PMC1443454

[B13] Okada M Nishimura F Yoshino H Kimura M Ogino T The J wave in accidental hypothermia J Electrocardiol 1983 16 23 28 10.1016/S0022-0736(83)80155-1 6833921

[B14] Vassallo S Delaney KA Hoffman RS Slater W Goldfrank LR A prospective evaluation of the electrocardiographic manifestations of hypothermia Acad Emerg Med 1999 6 1121 1126 10.1111/j.1553-2712.1999.tb00114.x 10569384

[B15] Jain A Wallis DE Shah K Blakeman BM Moran JF Electrocardiographic J waves after resuscitation from cardiac arrest Chest 1990 98 1294 1296 10.1378/chest.98.5.1294 2225988

[B16] Mattu A Brady WJ Perron AD Electrocardiographic manifestations of hypothermia Am J Emerg Med 2002 20 314 326 10.1053/ajem.2002.32633 12098179

[B17] Nolan J Soar J Images in resuscitation: the ECG in hypothermia Resuscitation 2005 64 133 134 10.1016/j.resuscitation.2004.11.027 15680518

[B18] Jacobs I Nadkarni V Bahr J Cardiac arrest and cardiopulmonary resuscitation outcome reports: update and simplification of the Utstein templates for resuscitation registries. A statement for healthcare professionals from a task force of the international liaison committee on resuscitation Resuscitation 2004 63 233 249 10.1016/j.resuscitation.2004.09.008 15582757

[B19] Bradley EH Herrin J Wang Y Barton BA Webster TR Mattera JA Roumanis SA Curtis JP Nallamothu BK Magid DJ McNamara RL Parkosewich J Loeb JM Krumholz HM Strategies for reducing the door-to-balloon time in myocardial infarction N Engl J Med 2006 355 2308 2320 10.1056/NEJMsa063117 17101617

[B20] Prineas R Crow R Blackburn H The Minnesota Code Manual of Electrocardiographic Findings 1982 Littleton, MA: John Wright-PSG

[B21] Bossaert L O'Connor RE Arntz HR Brooks SC Diercks D Feitosa-Filho G Nolan JP Hoek TL Walters DL Wong A Welsford M Woolfrey K Acute Coronary Syndrome Chapter Collaborators Part 9: acute coronary syndromes 2010 international consensus on cardiopulmonary resuscitation and emergency cardiovascular care patient with treatment recommendations Resuscitation 2010 81 E3175 E3212 10.1016/j.resuscitation.2010.09.001 20959169

[B22] Nolan JP Morley PT Vanden Hoek TL Hickey RW Kloeck WG Billi J Böttiger BW Morley PT Nolan JP Okada K Reyes C Shuster M Steen PA Weil MH Wenzel V Hickey RW Carli P Vanden Hoek TL Atkins D International Liaison Committee on Resuscitation Therapeutic hypothermia after cardiac arrest: an advisory statement by the advanced life support task force of the International Liaison Committee on Resuscitation Circulation 2003 108 118 121 10.1161/01.CIR.0000079019.02601.90 12847056

[B23] Binks AC Murphy RE Prout RE Bhayani S Griffiths CA Mitchell T Padkin A Nolan JP Therapeutic hypothermia after cardiac arrest: implementation in UK intensive care units Anaesthesia 2010 65 260 265 10.1111/j.1365-2044.2009.06227.x 20085568

[B24] Glusman A Hasan K Roguin N Contraindication to thrombolytic therapy in accidental hypothermia simulating acute myocardial infarction Int J Cardiol 1990 2 269 272 2394532 10.1016/0167-5273(90)90074-f

[B25] Norell MS Lyons JP Gardener JE Layton CA Balcon R Significance of 'reciprocal' ST segment depression: left ventriculographic observations during left anterior descending coronary angiography J Am Coll Cardiol 1989 13 1270 1274 10.1016/0735-1097(89)90299-4 2522957

[B26] Hoogendijk MG Potse M Coronel R Early repolarisation patterns: the good, the bad and the ugly Heart Rhythm 2012 9 230 231 10.1016/j.hrthm.2011.09.064 21952004

